# Regulation of Conidiogenesis in *Aspergillus flavus*

**DOI:** 10.3390/cells11182796

**Published:** 2022-09-07

**Authors:** He-Jin Cho, Sung-Hun Son, Wanping Chen, Ye-Eun Son, Inhyung Lee, Jae-Hyuk Yu, Hee-Soo Park

**Affiliations:** 1 School of Food Science and Biotechnology, Kyungpook National University, Daegu 41566, Korea; 2 Department of Molecular Microbiology and Genetics, University of Göttingen, 37077 Göttingen, Germany; 3 Department of Bio and Fermentation Convergence Technology, Kookmin University, Seoul 02707, Korea; 4 Department of Bacteriology, University of Wisconsin, Madison, WI 53706, USA; 5 Department of Systems Biotechnology, Konkuk University, Seoul 05029, Korea; 6 Department of Integrative Biology, Kyungpook National University, Daegu 41566, Korea

**Keywords:** velvet, asexual development, BrlA, AbaA, WetA, *Aspergillus flavus*

## Abstract

*Aspergillus flavus* is a representative fungal species in the *Aspergillus* section Flavi and has been used as a model system to gain insights into fungal development and toxin production. *A. flavus* has several adverse effects on humans, including the production of the most carcinogenic mycotoxin aflatoxins and causing aspergillosis in immune-compromised patients. In addition, *A. flavus* infection of crops results in economic losses due to yield loss and aflatoxin contamination. *A. flavus* is a saprophytic fungus that disperses in the ecosystem mainly by producing asexual spores (conidia), which also provide long-term survival in the harsh environmental conditions. Conidia are composed of the rodlet layer, cell wall, and melanin and are produced from an asexual specialized structure called the conidiophore. The production of conidiophores is tightly regulated by various regulators, including the central regulatory cascade composed of BrlA-AbaA-WetA, the fungi-specific velvet regulators, upstream regulators, and developmental repressors. In this review, we summarize the findings of a series of recent studies related to asexual development in *A. flavus* and provide insights for a better understanding of other fungal species in the section Flavi.

## 1. Introduction

The genus *Aspergillus* comprises more than 300 species, divided into 19 sections [1]. Among them, fungal species belonging to *Aspergillus* section Flavi are important for agriculture, biotechnology, the food industry, and human health [2]. For example, *Aspergillus flavus* is a major virulent fungus of maize, peanuts, and corn during crop harvesting, often coupled with the production of the most potent carcinogen in the nature aflatoxins, leading to great damage to crops, and economic loss [3,4]. In addition, *A. flavus* is the second most common agent of invasive aspergillosis [5,6]. Along with *A. flavus*, other species belonging to the section Flavi also produce various mycotoxins that have detrimental effects on humans [2]. Conversely, several species have beneficial applications in fermented foods, biotechnology, and pharmaceuticals [7]. For example, *A. oryzae* is used in traditional fermented foods, such as meju, soy sauce, miso, and sake [8,9]. *A. tamarii* and *A. alliaceus* are used for the enzymes they produce, such as amylases, protease, and pectin-degrading enzymes [10,11].

Most species in the genus *Aspergillus* reproduce through the production of spores released into the environment [12]. Some *Aspergillus* species produce sexual spores, but most *Aspergillus* species reproduce via asexual spores called conidia [13,14]. The asexual spore is carried on the conidiophore, an asexual specialized structure [15,16]. Unlike hyphae, conidia have a thick cell wall structure, which affords resistance to environmental conditions as well as protection from the immune system of the host organism [14,15]. In addition, conidia contain a variety of secondary metabolites, such as melanin and mycotoxins, which play key roles in pathogenesis and development [17,18]. The important processes of asexual spore formation, maturation, dormancy, and germination are controlled by several transcription factors and regulators [19,20]. Studies of asexual development in *Aspergillus* have focused on the model species *A. nidulans* [21]. Central regulators (BrlA, AbaA, and WetA), velvet regulators, upstream regulators (FlbA-E and FluG), and other components of the signaling pathway are involved in the regulation of asexual development [20]. The results obtained for *A. nidulans* have been applied to other *Aspergilli*; however, some of these regulators play diverse roles in members of Aspergilli. Recently, owing to advances in molecular and genetic technology, research on other species of *Aspergillus* has gradually expanded. In particular, whole-genome genetic information for representative strains in this section was presented in 2017 [22], and genomic information for 23 species of the *Aspergillus* section Flavi was recently studied, laying the foundation for customized research on various fungal species [23]. Based on the whole genomic information of strains belonging to *Aspergillus* section Flavi, genes encoding carbohydrate-active enzymes (CAZymes) and predicted secondary metabolite backbone genes have recently been analyzed [23]. These discoveries are expected to form the basis for the industrial production of enzymes or secondary metabolites.

*A.flavus*, a representative fungal species in Flavi, is more useful for understanding developmental processes in the section Flavi than is *A. nidulans* [3]. The species belonging to the *Aspergillus* section Flavi and *A. nidulans* have a common feature of generating an asexual specialized structure called conidiophore [12]. However, there are differences in the detailed morphology of asexual and sexual developmental structures, and metabolites. The main difference is that most species belonging to the *Aspergillus* section Flavi produce dark-colored sclerotia containing ascospore-bearing ascocarps [2], but *A. nidulans* produces cleistothecia surrounded by numerous Hülle cells [24,25]. In case of the morphology of conidiophores, *A. flavus* produces uni- or biseriate conidiophores, while *A. nidulans* produces biseriate conidiophores. For secondary metabolites, most species belonging to the *Aspergillus* section Flavi produce aflatoxins, but *A. nidulans* produces sterigmatocystin, which is a precursor of aflatoxins but not aflatoxins. As a result of several recent studies, new insights into the development and metabolism of *A. flavus* have been published [26,27]. Accordingly, this review aims to explore the distribution of developmental regulators in the strains belonging to Flavi and summarize the roles of developmental regulators in *A. flavus*. Developmental regulators related to asexual development are described in detail.

## 2. Distribution of Key Developmental Regulators in *Aspergillus*

**Figure 1:**
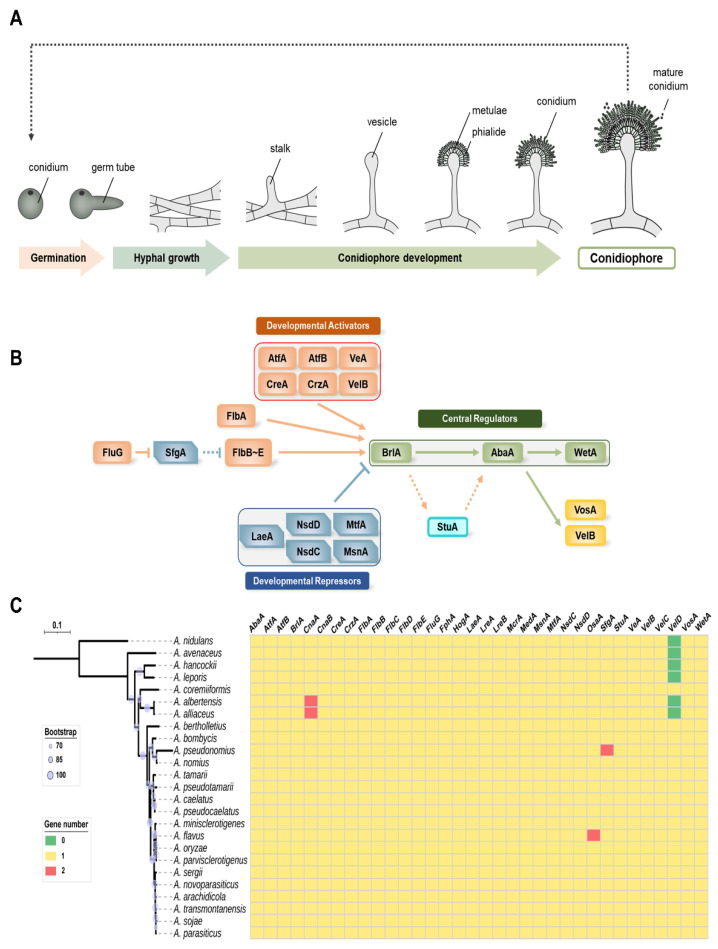
Asexual developmental regulators in *Aspergillus* spp. (**A**) A morphogenic model of conidiophore development of *A. flavus*. (**B**) A genetic model of the regulation of conidiogenesis in *A. flavus*. (**C**) Distribution of regulators involved in conidiogenesis in *Aspergillus* section Flavi. Distribution of 34 important regulators were investigated in 24 representative genomes from the section Flavi. The genomic data of *A. albertensis*, *A. alliaceus* CBS 536.65, *A. arachidicola*, *A. avenaceus* IBT 18842, *A. bertholletius* IBT 29228, *A. bombycis* NRRL 26010, *A. caelatus* CBS 763.97, *A. coremiiformis* CBS 553.77, *A. flavus* NRRL3357, *A. leporis* CBS 151.66, *A. minisclerotigenes* CBS 117635, *A. nomius* IBT 12657, *A. novoparasiticus* CBS 126849, *A. oryzae* RIB40, *A. parasiticus* CBS 117618, *A. parvisclerotigenus* CBS 121.62, *A. pseudocaelatus* CBS 117616, *A. pseudonomius* CBS 119388, *A. pseudotamarii* CBS 117625, *A. sergii* CBS 130017, *A. tamarii* CBS 117626, and *A. transmontanensis* CBS 130015 are obtained from Joint Genome Institute fungal genome portal MycoCosm (http://jgi.doe.gov/fungi, accessed on 4 August 2022). The genomic data of *A. hancockii* and *A. sojae* SMF134 were previously published [29,30]. The homologs were searched by BlastP using the regulators of *A. flavus* NRRL3357 as queries.

Several research groups have studied asexual development in *Aspergillus* spp. and have reported a variety of genes and proteins involved in the cycle of conidia formation (Figure 1A) [19,20,28]. We focused on 34 genes that are important for conidial formation, maturation, and dormancy (Figure 1B). The distribution of the 34 genes in 16 species of *Aspergillus* section Flavi is shown in Figure 1C. Most of the tested species of *Aspergillus* section Flavi contained one homolog of these genes. The genomes of *A. albertensis* and *A. alliaceus* contain two *cnaA* genes, encoding the catalytic subunit of calcineurin, whereas *A. pseudonomius* has two SfgA (suppressor of *fluG* A) homologs and *A. flavus* has two potential OsaA (orchestrator of sexual and asexual development A) homologs. Interestingly, VelD, the Velvet protein, has no homolog in *A. nidulans*, whereas most species belonging to *Aspergillus* section Flavi have it. However, VelD has not been found in several species, including *A. avenaceus*, *A. hanockii*, *A. leporis*, *A. albertensis*, and *A. alliaceus*. The roles of the studied genes are summarized in Table 1 and discussed below. Among 34 regulators, the roles of 26 genes were characterized in *A. flavus*. Other genes including *cnaA*, *cnaB*, *fphA*, *lreA*, *lreB*, *mcrA*, *medA*, and *osaA* have not been characterized, and further studies will be needed to understand *A. flavus* development.

### 2.1. Central Regulators of Conidiophore Production in Aspergillus

During asexual development, *Aspergillus* spp. generate conidiophores, which are composed of vesicles, one or two layers of sterigmata, and conidia [45]. *A. flavus* is biserate (metulae and phialide) or uniserate (phialide) species. The conidia of these species are produced by conidiogenous cell phialides [46]. Conidiophore formation in *Aspergillus* spp. is regulated by various regulators [20]. The expression of genes involved in conidiophore production is spatiotemporally regulated by three transcription factors: BrlA, AbaA, and WetA [13,47]. These transcription factors are highly conserved in most *Aspergillus* species [19]. Studies on these transcription factors have been mainly conducted in *A. nidulans* and *A. fumigatus*, but not much research has been done on other species of *Aspergillus* [47,48,49,50]. In *A. flavus*, the role of WetA, but not BrlA and AbaA, has been characterized [44,51,52]. Therefore, the roles of BrlA and AbaA were described based on our current study and studies in other species.

#### 2.1.1. BrlA

**Figure 2:**
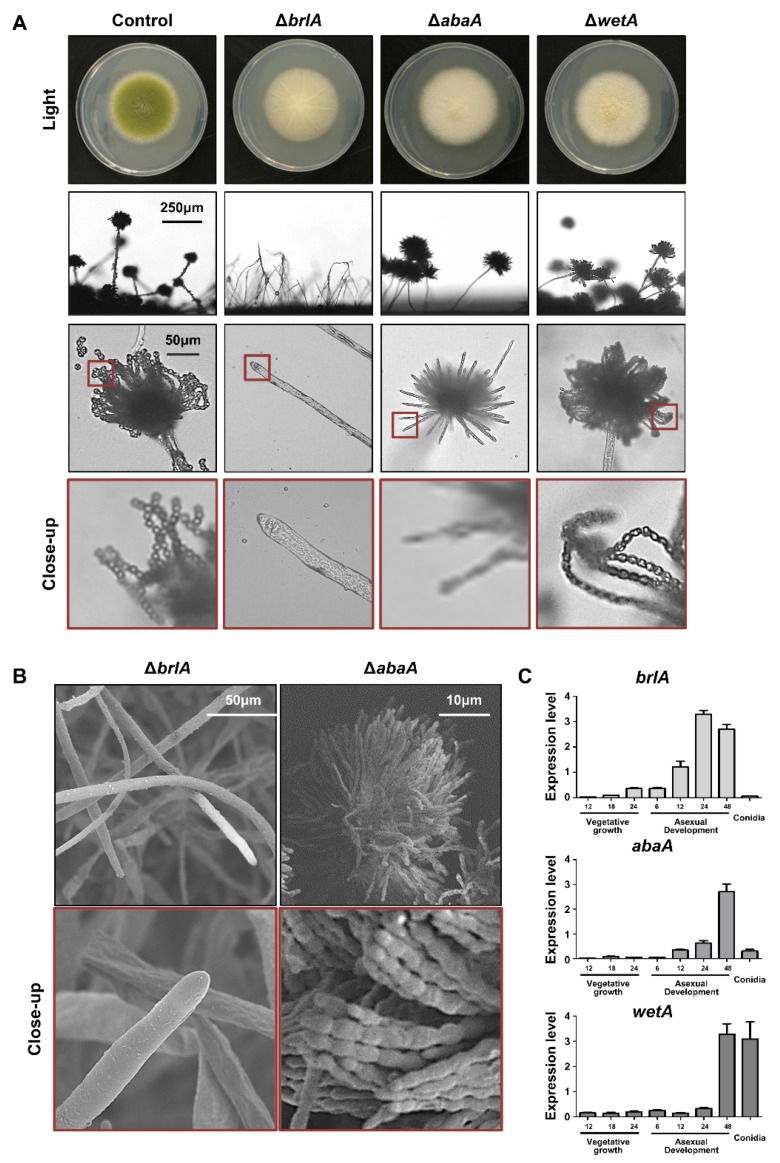
Summary of the central regulators in *A. flavus*. (**A**) Phenotypes of Δ*brlA*, Δ*abaA*, and Δ*wetA* mutant strains. Colony phenotypes of Δ*brlA*, Δ*abaA*, and Δ*wetA* strains point-inoculated on solid glucose minimal medium with 0.1% yeast extract media and grown at 37 °C (Upper). Morphology of Δ*brlA*, Δ*abaA*, and Δ*wetA* conidiophores observed under a light microscope at 48 h after inoculation onto solid MMYE media at 37 °C (Bottom). (**B**) Scanning electron micrographs of Δ*brlA* and Δ*abaA* strains. (**C**) mRNA levels of *brlA*, *abaA*, and *wetA* during *A. flavus* life cycle. Samples for RT-qPCR analysis were collected from 12, 18, and 24 h of vegetative growth; 6, 12, 24, and 48 h of asexual development, and in conidia.

The *brlA* (bristle A) gene was first identified through mutational analysis of asexual development in *A. nidulans* [53]. The *brlA* mutants are aconidial, and these mutant strains exhibit a bristle-like phenotype [47]. This aconidial phenotype was similar to that of *A. flavus*. As shown in Figure 2A, the *brlA* mutant forms white colonies and cannot produce a proper conidiophore. These results are similar to those of the scanning electron microscopes image; where it can be seen that the *brlA* deletion mutants formed a hyphal tip but did not form a conidiophore (Figure 2B). In *A. oryzae*, the *brlA* mutant exhibits no conidiation and increased hyphal growth [54,55], suggesting that the function of *brlA* in conidiophore production is conserved in *Aspergillus* species.

**Figure 3:**
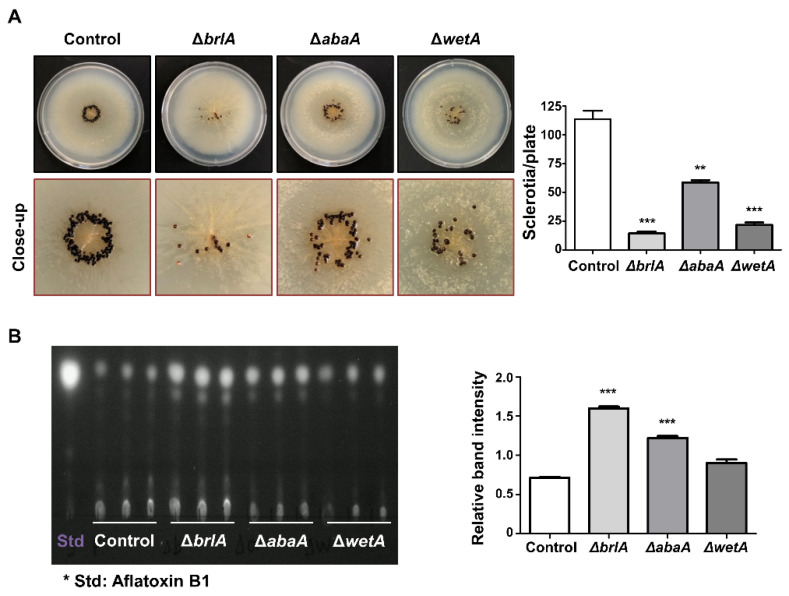
Roles of the central regulators in *A. flavus* sclerotia and aflatoxin production. (**A**) Ethanol-washed colony photographs of Δ*brlA*, Δ*abaA*, and Δ*wetA* strains grown on solid minimal media with 1% glucose and 0.1% yeast extract (MMYE) for 7 days. Quantitative analysis of sclerotia of these strains. (**B**) Image of thin-layer chromatography (TLC) of aflatoxin B1 from Δ*brlA*, Δ*abaA*, and Δ*wetA* strains under dark conditions. To extract aflatoxin B1 from each strain, about 10^7^ conidia were inoculated into liquid complete media and incubated for 7 days at 30°C in dark condition. To extract aflatoxin B1, chloroform was used. The samples were spotted onto a TLC silica plate, and the plate was placed into a chamber containing chloroform: acetone (9:1, *v*/*v*). Densitometry of the TLC analysis results. Statistical differences between control and mutant strains were evaluated using Student’s unpaired *t*-tests. Data are reported as the mean ± standard deviation. ** *p* < 0.01, *** *p* < 0.001.

BrlA is a key transcription factor for the initiation of conidiophore formation, which contains a C_2_H_2_ zinc finger DNA-binding domain [56,57,58]. In *A. nidulans*, BrlA is highly expressed during the initiation of conidiation and regulates genes containing the BrlA response element (BRE, 5′-CAAGGGG-3′) at the promoter region in the target genes (e.g., *abaA*) [57]. Similar to *A. nidulans*, *brlA* mRNA was highly expressed during the early phase of conidiation and was not detected in the conidia of *A. flavus* (Figure 2C). BrlA has been reported to affect sexual development and secondary metabolism [45,59]. For example, the *brlA* mutant cannot produce cleistothecia in *A. nidulans* [45]. In *A. flavus*, sclerotia production was shown to be decreased in the *brlA* mutant (Figure 3A), implying that BrlA plays a diverse role in sexual reproduction in *Aspergillus* spp. The production of aflatoxin was increased in the *brlA* mutant strains (Figure 3B). These results suggest that BrlA is conserved in *Aspergillus* conidiation and is involved in fungal reproduction and secondary metabolism.

#### 2.1.2. AbaA

AbaA is a key regulator of gene expression during the middle phase of conidiation [60,61]. The typical phenotype of the *abaA* mutant is an abacus structure that incompletely differentiates phialides from the conidial chain in *A. nidulans* [60]. This phenotype has also been observed in other *Aspergillus* species, such as *A. fumigatus* and *A. oryzae* [49,54]. In *A. flavus*, the *abaA* mutant also exhibited white colonies and abacus-like phialides (Figure 2), similar to what is found in other species. AbaA also affects the proper production of sclerotia and aflatoxin B (Figure 3).

AbaA is a transcription factor containing a TEA/ATTS domain [43]. The *abaA* gene was highly expressed during the middle and late phases of conidiation but not in conidia (Figure 2C). It has been reported that AbaA recognizes the DNA sequence 5′-CATTCY-3′ (Y is a pyrimidine), called AbaA response element (ARE) [62]. AREs are present in the promoter regions of AbaA target genes such as *wetA*, *vosA*, and *rodA* [63]. In *A. flavus*, these target genes also contain AREs in their promoter regions, and mRNA expression of these target genes is regulated by AbaA. These molecular, bioinformatics, and morphological results strongly support the idea that the role of AbaA is conserved for regulating the middle phase of conidiation in *Aspergillus* species.

#### 2.1.3. WetA

WetA is a DNA-binding transcription factor with an ESC1/WetA-related domain that directly or indirectly regulates genes involved in conidial formation, wall integrity, and metabolism [64]. The *wetA* deletion mutant exhibits a white colony, but the shape of the conidiophore is similar to that of the wild-type (Figure 2). However, when observed under a transmission electron microscope, there is a significant difference in spore size and wall integrity [44]. In addition, the amount of β-glucan increased, but the content of trehalose decreased in the mutant spores [44]. The RNA-seq results demonstrate that WetA coordinates the mRNA expression of genes related to trehalose, chitin, glucan, and melanin metabolic pathways [44]. The role of WetA is highly conserved among *Aspergillus* species. Wu and colleagues found a potential WetA response element (WRE) via the ChIP-seq in *A. nidulans*, and WREs can be found in the promoter regions of potential WetA target genes in *A. fumigatus* and *A. flavus* [52]. These results suggest that WetA is a key regulator of the gene regulatory network in *Aspergillus* conidia. Similar to other central regulators, the loss of *wetA* decreased sclerotia production but increased aflatoxin B1 production (Figure 3).

### 2.2. Upstream Regulators in Asexual Development

For the formation of conidiophores to start in hyphae, developmental competence, the ability to respond to developmental cues, must be acquired [65,66]. Until developmental competence is acquired, upstream developmental activators occupy the promoter region of *brlA*, but conidiation does not begin because several repressors bind the promoter of *brlA* and block the role of upstream developmental activators [67]. After developmental competence is acquired, repressors are removed from the *brlA* promoter and *brlA* expression is induced to initiate conidiation [20]. Therefore, to understand the initiation of asexual development, it is important to understand the upstream activators or repressors and the signaling pathways involved in the initiation of *brlA*.

#### 2.2.1. Developmental Activators

The *fluffy* genes are well-studied genes that encode developmental activators involved in the expression of *brlA* in *A. nidulans* [16]. Mutational analysis identified six *fluffy* genes: *fluG*, *flbA*, *flbB*, *flbC*, *flbD*, and *flbE* [20,68]. The loss of function mutations in any one of these genes results in a fluffy colony.

FluG contains two domains: an amidohydrolase domain in the N-terminal region and a γ-glutamyl ligase region in the C-terminal region [69,70,71]. FluG is involved in the production of dehydroaustinol, a diffusible activator of conidiation in *A. nidulans* [72,73]. Therefore, FluG is a key gene in the initiation of conidiation. Unlike *A. nidulans*, Δ*fluG* mutants can produce asexual spores of *A. flavus* [35]. The deletion of *fluG* causes reduced conidial production and *brlA* expression but increased sclerotia production, suggesting that FluG may act as a balancer for asexual and sexual development [35]. Further studies have found that FluG can interact with VelB or LaeA, which then controls conidia and sclerotia production [74].

FlbA is one of the regulators of G protein signaling (RGS) proteins that are involved in the G protein signaling pathway [75,76]. FlbA affects fungal development and aflatoxin production in *A. flavus* [34]. The deletion of *flbA* decreased conidia production and *brlA* expression. In addition, the Δ*flbA* mutant strains show decreased pathogenicity and toxigenicity [34]. In *A. nidulans*, FlbA is involved in the FadA-cAMP/PKA pathway; however, in *A. flavus*, FlbA does not affect the FadA signaling pathway or the regulation of intracellular cAMP concentration [34].

**Figure 4:**
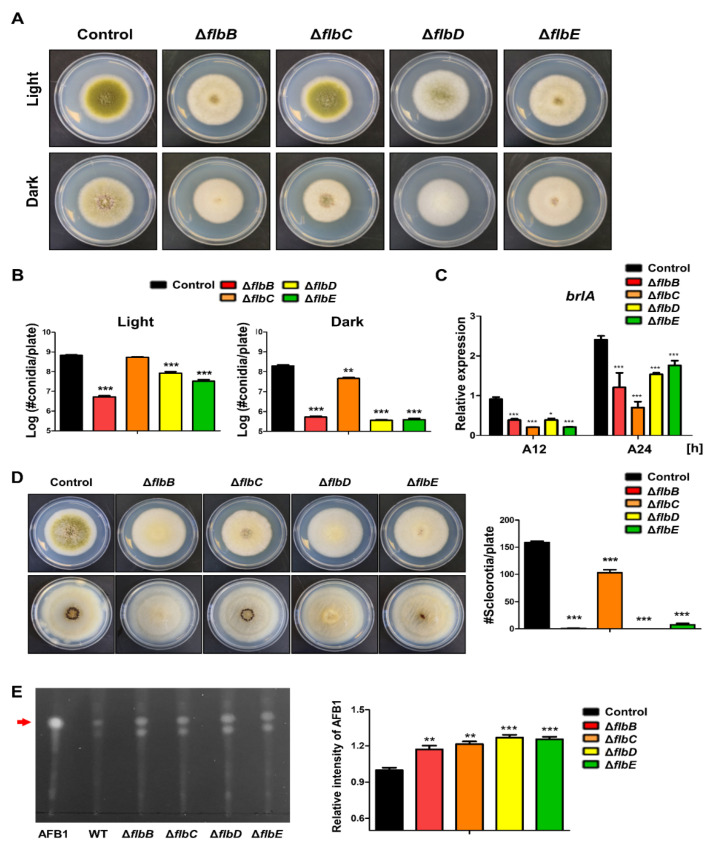
Roles of the *fluffy* genes in *A. flavus.* (**A**) Colony morphology of Δ*flbB*, Δ*flbC*, Δ*flbD*, and Δ*flbE* mutant strains. (**B**) Quantitative analysis of the number of conidia per plate shown in (**A**). (**C**) mRNA level of brlA in wild-type and mutant strains. (**D**) Ethanol-washed colony photographs of *fluffy* mutant strains. (**E**) Image of TLC of aflatoxin B1 from *fluffy* mutant strains. Densitometry of TLC analysis results. Statistical differences between control and mutant strains were evaluated using Student’s unpaired *t*-tests. Data are reported as the mean ± standard deviation. * *p* < 0.05, ** *p* < 0.01, and *** *p* < 0.001.

*flbB*–*flbD* are fluffy genes that encode DNA-binding transcription factors [67]. The roles of FlbB–FlbE have been studied in *A. nidulans*, *A. fumigatus*, and *A. oryzae*, and these transcription factors are key components for conidiophore initiation and *brlA* expression [54,67,77,78,79,80,81,82,83]. In *A. flavus*, the roles of FlbB–FlbE are similar to those in other *Aspergilli*, but they are slightly different from those in *A. nidulans*. In *A. nidulans*, the loss of *flbB*~*flbE* has been shown to cause delayed or decreased production of conidiophores and *brlA* expression. In *A. flavus*, each mutant strain of *flbB, flbD*, and *flbE* can produce a small number of asexual spores, the *flbC* mutant produces asexual spores similar to the wild-type strains in light conditions (Figure 4). In terms of *brlA* expression, all *flbB*~*flbE* strains express *brlA* to a lesser degree than the wild-type (Figure 4C), which suggests that *flbB*~*flbE* are important for the initiation of conidiophore production.

The functions of FlbB–FlbE in asexual spore formation are conserved in both *A. nidulans* and *A. flavus* (Table 2). However, sexual development has slightly different functions. In *A. nidulans*, the production of sexual fruiting bodies, called cleistothecia, was increased in the *flbC* mutant, but the amount of sclerotia was decreased in *A. flavus*. Overall, although their roles in sexual development and secondary metabolites are somewhat different, they are similar in asexual reproduction in *Aspergillus* species.

#### 2.2.2. Velvet Regulators and LaeA

Velvet proteins contain a fungus-specific DNA-binding domain, called the velvet domain, which is a transcription factor that regulates multiple genes [84,85]. In most *Aspergillus* species, the velvet protein family consists of four proteins, including VeA, VelB, VelC, and VosA, but there are five proteins in *A. flavus* and other Flavi species (Figure 1B) [43]. Velvet proteins also form homo- or heterocomplexes that coordinate fungal reproduction and secondary metabolism [86,87]. VeA has been studied in several fungal species and plays an important role in fungal development and secondary metabolism [86,88]. In *A. flavus*, the deletion of *veA* results in decreased conidia formation, the absence of sclerotia, and aflatoxin B formation [37,74]. VeA regulates lipid degradation in seeds, which affects plant pathogenesis [37]. VeA also forms a complex with VelB and LaeA, which controls sexual development and aflatoxin production [37,74]. It has been reported that VelB, a partner protein of VeA, functions similarly to VeA in sexual reproduction, conidiophore formation, and aflatoxin production. However, VelB also interacts with other proteins, such as FluG or VosA, and these complexes are involved in other processes. It has been predicted that VelB functions together with FluG during asexual spore formation [74]. The VelB-VosA complex governs conidia maturation and stress response in conidia [43]. VelC and VelD do not appear to play a significant role in asexual spore formation, but VelD affects the aflatoxin formation process [43]. VosA is a key regulator for the viability of spores in *A. nidulans*. As mentioned above, VosA plays a key role in spore maturation and dormancy after conidia formation by binding to VelB. The deletion of *vosA* or *velB* causes increased stress sensitivity and decreases the amount of trehalose in *A. flavus* conidia [43]. VelB and VosA are representative transcription factors that regulate the mRNA expression of spore-specific genes, together with WetA, after spore formation [51]. These regulators control the genes associated with chitin and beta-glucan biosynthesis, trehalose synthesis, and secondary metabolism in *A. nidulans* and *A. flavus* conidia [51,52].

LaeA is a putative methyltransferase that has been identified as a forward genetic screen in *A. nidulans* [89]. It has been reported that the main function of LaeA in various fungal species is the production of sterigmatocystin and other secondary metabolites [90]. Importantly, as mentioned above, LaeA interacts with other proteins to form complexes, such as VeA-VelB-LaeA or VeA-VelB-LaeA-FluG, which coordinate aflatoxin production and fungal development [54,66]. We predicted that members of these complexes would perform similar functions in conidia production in *A. flavus*, but the phenotypes of each mutant were slightly different. For example, the deletion *veA* or *velB* led to decreased conidia production, but the *laeA* mutant exhibited increased conidia production, and the morphology of *laeA* mutant conidiophores was abnormal [91]. These results support the idea that LaeA and VeA/VelB play different roles in the asexual development of *A. flavus* compared with *A. nidulans* [74].

### 2.3. Other Key Regulators for Asexual Development

#### 2.3.1. AtfA and AtfB

AtfA and AtfB are basic leucine zipper (bZIP) transcription factors that affect fungal development, metabolism, and stress responses [92,93,94]. Importantly, AtfA is one of the components of the high-osmolarity glycerol (HOG) MAPK cascade, which is a key signaling pathway for hyperosmotic and oxidative stress response [31]. In *A. flavus*, the absence of *atfA* or *atfB* leads to decreased fungal growth, conidiation, sclerotia production, and aflatoxin B1 production. In addition, AtfA and AtfB, but not osmotic and alkali stress, are required for the oxidative stress response [31]. These results provide insights into the roles of AtfA and AtfB in conidiophore production and stress response in *A. flavus*.

#### 2.3.2. CreA

Carbon is the main energy source, and carbon catabolite repression (CCR) is required for the regulation of development and metabolic processes in fungi [95]. CreA contains the Cys_2_-His_2_ zinc finger domain and acts as a major transcriptional repressor in *A. nidulans* [96,97]. Fasoyin et al. characterized the function of CreA and found that the deletion of *creA* caused the production of abnormal conidiophores, decreased *brlA* and *abaA* mRNA expression, and the production of aflatoxin in *A. flavus* [32]. The overexpression of *creA* increases the number of conidia and aflatoxin B1 production in maize seeds. These results suggest that CreA acts as a positive regulator of asexual development in *A. flavus*.

#### 2.3.3. CrzA

The calcineurin-Crz1 signaling pathway plays a key role in the stress response, development, and virulence of pathogenic fungi [98]. Crz1/CrzA is a C_2_H_2_-type transcription factor that is a key target of calcineurin in yeast and filamentous fungi [99,100,101,102]. In *A. flavus*, the loss of *crzA* resulted in reduced conidial production and abnormal conidiophore production [33]. The mRNA levels of *brlA*, *abaA*, and *wetA* significantly decreased in the *crzA* deletion mutant strains during asexual development. Fungal growth and sclerotia production were decreased in *crzA* deletion mutants [82]. Although the *crzA* mutant strain was generated, it was difficult to generate *cnaA* or *cnaB* deletion mutants. In addition, it was difficult to generate calcineurin mutants in other *Aspergillus*, or calcineurin mutant strains grow very slowly [103], implying that the calcineurin-Crz1 signaling pathway is crucial for fungal growth and the initiation of asexual and sexual development.

#### 2.3.4. HogA (SakA)

The high-osmolarity glycerol (HOG) pathway is necessary for tolerance to a variety of environmental stresses and immune responses in the host system [104,105]. HogA (also called SakA) is a key kinase in the HOG pathway in fungi [106]. In *A. flavus*, HogA is a key factor for osmotic stress response, aflatoxin B1 production, and virulence [36]. HogA also affects conidiation. The deletion of *hogA* led to decreased conidiophore production under normal conditions but increased the number of conidia under osmotic stress conditions or in seeds, suggesting that HogA plays a diverse role in asexual development depending on the environmental conditions.

#### 2.3.5. MsnA

MsnA is an ortholog of *S. cerevisiae* Msn2, which contains a C_2_H_2_-type zinc-finger domain [107]. MsnA is associated with the HOG pathway, which is crucial for stress response in *A. nidulans* [89]. In *A. flavus* and *A. parasiticus*, MsnA plays a similar role in the stress response [38]. In addition, the deletion of *msnA* decreased fungal growth but increased conidia production [38]. MsnA also affects secondary metabolism, and the *msnA* deletion mutant produces more aflatoxin B1 and kojic acid. MsnA plays a key role in both stress response and fungal development.

#### 2.3.6. MtfA

MtfA is a master transcription factor for secondary metabolite production in *Aspergillus* species [108,109]. This protein contains a C_2_H_2_ DNA-binding domain that governs the production of secondary metabolites. In *A. flavus*, *mtfA* overexpression mutants cannot produce aflatoxin and can affect the production of several secondary metabolites [39]. For asexual development, the deletion of *mtfA* causes increased conidia production and induces *brlA* expression, but the overexpression of *mtfA* causes a decrease in the number of conidia and *brlA* expression. These results strongly support the idea that MtfA negatively regulates *A. flavus* asexual development.

#### 2.3.7. NsdC and NsdD

NsdC (never in sexual development) is a transcription factor containing a C_2_H_2_ zinc finger-type DNA-binding domain [110]. NsdC is a gene identified through genetic screening, together with NsdD, which contains a GATA-type zinc finger domain. *nsdC* or *nsdD* deletion mutants cannot produce sexual structures in *A. nidulans* [111]. NsdD acts as a repressor of *brlA* expression, which is exerted by directly binding to the promoter of *brlA* in *A. nidulans* [112]. Both genes affect asexual development and aflatoxin production in *A. flavus* [40]. The deletion of *nsdC* or *nsdD* causes the production of abnormal conidiophores, with shortens stipes and alters conidial heads. In addition, the Δ*nsdC* and Δ*nsdD* mutants exhibited abnormal increases in the expression of *brlA* and *abaA*. In the case of NsdC, the Δ*nsdC* strain produced more conidiophores than the control strain. RNA-seq analysis demonstrated that NsdC affects the mRNA expression of asexual developmental genes such as *brlA*, *abaA*, *wetA*, *vosA*, and *yA* [113]. Lee and colleagues found that *nsdD* deletion mutants produced abnormal conidiophores in liquid submerged cultures and formed abundant conidiophores embedded in agar [112]. Overall, these results strongly suggest that NsdC and NsdD are key negative regulators of conidiation and may directly regulate *brlA* expression in *A. flavus*.

#### 2.3.8. SfgA

SfgA (suppressors of *fluG*), which contains a Zn_2_Cys_6_ DNA-binding domain, is a negative regulator of asexual development functioning downstream of FluG in *A. nidulans* [114,115]. The role of SfgA in *A. flavus* has been examined by Yuan et al. [41]. Phenotypic analyses of the *sfgA* deletion or overexpression mutant demonstrated that SfgA plays a similar role in asexual development in *Aspergillus* species. The *sfgA* deletion mutant can produce asexual developmental structures in liquid submerged culture, implying that SfgA functions as a repressor of conidiophore production. Genetic analysis has revealed that SfgA is a downstream transcription factor of FluG. However, it is unclear whether SfgA is an upstream regulator of FlbB-E, and further studies are needed to confirm this.

#### 2.3.9. StuA

The *stuA* (*stunted*) gene encodes a protein that is a member of the APSES transcription factors (Asm1p, Phd1p, Sok2p, Efg1p, and StuAp) [116,117] In *A. nidulans*, StuA controls the mRNA expression of *brlA* and *abaA,* which then modulate asexual sporulation [117]. In *A. fumigatus*, *stuA* mRNA is expressed after the acquisition of developmental competence. In addition, the Δ*stuA* mutant strains produced abnormal conidiophores and a small number of conidia, suggesting that StuA is crucial for proper conidiophore production in *A. fumigatus* [118]. Yao et al. demonstrated that StuA plays a key role in fungal development and secondary metabolism in *A. flavus* [42]. The *stuA* mutants exhibited impaired conidiophore production. StuA affects *brlA* and *abaA* expression, which affects the expression of downstream genes, thereby resulting in conidiophore formation. Moreover, the absence of *stuA* results in defects in conidiation and aflatoxin production in peanut and maize seeds. Overall, these results suggested that StuA is a key regulator of conidiation and metabolism in *A. flavus*.

## 3. Conclusions

With recent advances in next-generation sequencing, the genomes of various *Aspergillus* species have been reported, but knowledge based on biological and molecular research is required to understand fungal biology in detail. For this purpose, *A. nidulans* was primarily used as a model fungus for all *Aspergillus* species. However, due to the development of genetic and molecular techniques in recent decades, a variety of studies have been conducted on *A. flavus*, which have provided useful information for understanding the fungi belonging to *Aspergillus* section Flavi. *A. flavus* has been studied mainly due to its toxin production and pathogenicity, but to understand all of this, it is necessary to understand asexual development, the major reproductive mode of *Aspergillus* spp. In this review, the functions of the various developmental regulators involved in asexual reproduction were described and discussed. With these developmental regulators, the function of other transcription factors was published (Table 3). However, most studies have derived functions based on phenotypic analyses. In the near future, it will be necessary to systematically understand asexual development using new techniques such as transcriptomic, metabolomic, and metagenomic analyses. It is hoped that this will provide insights to understand fungal development in *A. flavus* and other fungal species in the *Aspergillus* section Flavi.

## Figures and Tables

**Table 1:** The function of the major developmental regulators in studied *A. flavus* conidiation.

Genes	Domain(s)	Description	Ref.
AbaA	TEA/ATTS	Regulator for sterigmata formation	-
AtfA	bZIP	Developmental activator	[31]
AtfB	bZIP	Developmental activator	[31]
BrlA	C_2_H_2_ zinc finger	Initiator for conidiogenesis	-
CreA	C_2_H_2_ zinc finger	Developmental activator	[32]
CrzA	C_2_H_2_ zinc finger	Developmental activator	[33]
FlbA	RGS	Upstream developmental activator	[34]
FlbB	Basic leucine zipper	Upstream developmental activator	-
FlbC	C_2_H_2_ zinc finger	Upstream dev. activator	-
FlbD	Myb-like DNA binding	Upstream dev. activator	-
FlbE	-	Upstream dev. activator	-
FluG	Amidohydrolase and GS	Upstream dev. activator	[35]
HogA	Protein kinase	Modulator for conidiation	[36]
LaeA	SAM	Developmental repressor	[37]
MsnA	C_2_H_2_ zinc finger	Developmental repressor	[38]
MtfA	C_2_H_2_ zinc finger	Developmental repressor	[39]
NsdC	C_2_H_2_ zinc finger	Developmental repressor	[40]
NsdD	GATA-type zinc-finger	Developmental repressor	[40]
SfgA	Zn_2_Cys_6_	Developmental repressor	[41]
StuA	APSES	Modulator for conidiophore formation	[42]
VeA	Velvet	Developmental activator	[37]
VelB	Velvet	Developmental activator Regulator for conidial maturation	[43]
VelC	Velvet	-	[43]
VelD	Velvet	-	[43]
VosA	Velvet	Regulator for conidial maturation	[43]
WetA	ESC1/WetA-related	Regulator for conidial maturation	[44]

bZIP, basic leucine zipper; RGS, regulator of G protein signaling; SAM, S-adenosyl-l-methionine; GS, glutamine synthetase; and APSES, Asm1p, Phd1p, Sok2p, Efg1p, and StuAp.

**Table 2:** Comparison of the developmental phenotypes of the Δ*flb* mutant strains in *A. nidulans* and *A. flavus*.

Genes		Asexual Development		Sexual Structure Formation	Ref.
		Conidia Formation	*brlA* Expression		
FlbB	Δ*AniflbB*	decrease	absence	not determined	[77]
	Δ*AflflbB*	decrease	decrease	decrease	-
FlbC	Δ*AniflbC*	decrease	delay	increase	[80]
	Δ*AflflbC*	decrease	decrease	decrease	-
FlbD	Δ*AniflbD*	decrease	delay	decrease	[79]
	Δ*AflflbD*	decrease	decrease	decrease	-
FlbE	Δ*AniflbE*	decrease	delay	not determined	[78]
	Δ*AflflbE*	decrease	decrease	decrease	-

**Table 3:** The function of transcription factors related to *A. flavus* conidiation.

Genes	Domain(s)	Phenotype of Deletion Mutant	Ref.
AflR	Zn_2_Cys_6_ domain	Decrease conidiophore production	[119]
AreA	GATA zinc finger domain	Decrease conidiophore production and *brlA* expression	[120]
Cti6	PHD domain	Decrease conidiophore production and *brlA* expression	[121]
Hbx1	Homeodomain	Loss of conidiophore, decrease *brlA* expression	[122]
RafA	APSES	Decrease conidiophore production, increase *brlA* expression	[42]
RsrA	C_2_H_2_ zinc finger	Decrease conidiophore production	[123]
Rum1	PHD domain	Increase conidiophore production and *brlA* expression	[124]
Skn7	Heat-shock transcription factor-like DNA-binding domain	Abnormal conidiophore, decrease conidiophore production	[125]
ZcfA	Zn_2_Cys_6_ domain	Increase conidiophore production	[126]

PHD, plant homeodomain; APSES, Asm1p, Phd1p, Sok2p, Efg1p, and StuAp.

## Data Availability

Not applicable.
